# Cas12a-Based On-Site and Rapid Nucleic Acid Detection of African Swine Fever

**DOI:** 10.3389/fmicb.2019.02830

**Published:** 2019-12-10

**Authors:** Jing Bai, Haosi Lin, Haojian Li, Yang Zhou, Junshan Liu, Guorui Zhong, Luting Wu, Weifan Jiang, Hongli Du, Jinyi Yang, Qingmei Xie, Lizhen Huang

**Affiliations:** ^1^School of Biology and Biological Engineering, South China University of Technology, Guangzhou, China; ^2^College of Food Science, South China Agricultural University, Guangzhou, China; ^3^College of Animal Science, South China Agricultural University, Guangzhou, China

**Keywords:** CRISPR/Cas12a, African swine fever, on-site detection, lateral flow strip, nucleic acid detection

## Abstract

The mortality rate of hemorrhagic African swine fever (ASF), which targets domestic pigs and wild boars is caused by African swine fever virus (ASFV), can reach 100%. Since the first confirmed ASF outbreak in China on 3 August 2018, 156 ASF outbreaks were detected in 32 provinces. About 1,170,000 pigs were culled in order to halt further spread. There is no effective treatment or vaccine for it and the present molecular diagnosis technologies have trade-offs in sensitivity, specificity, cost and speed, and none of them cater perfectly to ASF control. Thus, a technology that overcomes the need for laboratory facilities, is relatively low cost, and rapidly and sensitively detects ASFV would be highly valuable. Here, we describe an RAA-Cas12a-based system that combines recombinase aided amplification (RAA) and CRISPR/Cas12a for ASFV detection. The fluorescence intensity readout of this system detected ASFV p72 gene levels as low as 10 aM. For on-site ASFV detection, lateral-flow strip readout was introduced for the first time in the RAA-Cas12a based system (named CORDS, Cas12a-based On-site and Rapid Detection System). We used CORDS to detect target DNA highly specifically using the lateral-flow strip readout and the assay displayed no cross-reactivity to other 13 swine viruses including classical swine fever (CSF). CORDS could identify the ASFV DNA target at femtomolar sensitivity in an hour at 37°C, and only requires an incubator. For ease of use, the reagents of CORDS were lyophilized to three tubes and remained the same sensitivity when stored at 4°C for at least 7 days. Thus, CORDS provide a rapid, sensitive and easily operable method for ASFV on-site detection. Lyophilized CORDS can withstand long-term transportation and storage, and is ready for field-based applications.

## Introduction

African swine fever (ASF) is a highly contagious hemorrhagic disease of domestic pigs and wild boars. ASF is caused by a large, complex double-stranded DNA virus, the African swine fever virus (ASFV) (Galindo and Alonso, 2017). The disease shares similar clinical signs with classical swine fever (CSF) and several other swine diseases (Oura et al., 2013), making its diagnosis difficult, especially outside of a diagnostics laboratory By 5 September 2019, 156 outbreaks were detected in China and 1,170,000 pigs were culled in an effort to halt further spread (Food and Agricultural Organization of the United Nations, 2019). There is no effective treatment or vaccine against ASF yet, so controlling ASF mainly relies on animal slaughter and sanitary measures (Galindo and Alonso, 2017; Dixon et al., 2019). Therefore, accurate and timely diagnosis of ASF infections is crucial for controlling epidemics.

The recommended gold standard for diagnosis according to the World Organization for Animal Health (OIE) is virus isolation. But this is time-consuming and labor-intensive, and virus isolation is not a method applicable to disease monitoring and control (Oura et al., 2013). The antigen enzyme-linked immunosorbent assay is a rapid and convenient method for detecting ASFV antigens (Oura et al., 2013). However, it lacks sensitivity in subacute cases or for early-stage infections and has poor specificity because it cannot correctly identify different viral strains (Oura et al., 2013; Boonham et al., 2014). Hence, the molecular tools for detecting ASFV, such as polymerase chain reaction (PCR) and reverse-transcriptase (RT)-PCR, have become popular because of their sensitivity and specificity for ASF diagnosis (Oura et al., 2013). Nevertheless, such methods are not suitable for on-site situations (e.g., on farms) or for rapid viral detection as they require thermal cycling instruments and skilled operators (Ikeno et al., 2013; Boonham et al., 2014; Khodakov et al., 2016). Isothermal amplification techniques, such as recombinase polymerase amplification (RPA), loop mediated isothermal amplification and cross-priming amplification, have been successfully used to detect ASFV (Oura et al., 2013). Recently, many novel isothermal amplification assays in combination with immunochromatographic strips have also been developed for on-site detection of ASFV (Gao et al., 2018; Miao et al., 2019). With no need for instruments and skilled operators, isothermal amplification has become a promising on-site detection method. But like other amplification technologies, its resolution depends on the binding specificity between the primers and templates, which can limit their accuracy (Boonham et al., 2014; He et al., 2019).

With its ability to accurately recognize specific sequence, the CRISPR/Cas system holds promise as a detection method (Huang et al., 2018; Sashital, 2018; Li et al., 2019). The *trans*-cleavage activity of the recently reported Cas12a, Cas13a, and Cas14 systems in particular, helps them first bind to the target specifically and then amplify the binding signal by cutting the dual-labeled probe in a nonspecific way (Gootenberg et al., 2017; Chen et al., 2018; Harrington et al., 2018; Qian et al., 2019). New nucleic acid detection technologies for different purposes using the CRISPR/Cas12a and Cas13a systems, such as DETECTR, SHERLOCK and HOLMES etc., have extremely high sensitivities and specificities (single base resolution), and have been developed in combination with other gene amplification methods, including PCR and RPA (Gootenberg et al., 2017, 2018; Chen et al., 2018; Myhrvold et al., 2018; Santos et al., 2018; Shan et al., 2019). Cas12-based DETECTR and HOLMES need fluorescent detection equipment and therefore are not suitable for field-based tests (Chen et al., 2018; Li et al., 2018; Santos et al., 2018). In contrast, when Cas13-based SHERLOCK is combined with an immunochromatographic strip, it becomes a good choice for on-site detection (Gootenberg et al., 2018; Myhrvold et al., 2018). However, Cas13-based system like SHERLOCK, both the target and probe are RNA, which increases the detection costs and reduces the assay’s stability. Moreover, the RNA probe can generate false positive results because RNase is ever present and RNA is generally unstable (Gootenberg et al., 2017, 2018; Myhrvold et al., 2018; Sashital, 2018; Shan et al., 2019). The newly reported naked-eye gene detection platform based on the CRISPR/Cas12a/13a system also needs a centrifugal machine and whole blood samples can interrupt the naked-eye detection (Yuan et al., 2019).

Therefore, the Cas12a-based system using a single-stranded DNA probe is more suitable for viral detection where limited conditions for on-site detection exist such as on farms (Sashital, 2018). In this study, we combined Cas12a with recombinase aided amplification (RAA) and an immunochromatographic lateral flow strip to develop a test strip method, termed CORDS (Cas12a-based On-site and Rapid Detection System) for ASFV on-site detection. CORDS can detect the ASFV target at a sensitivity of 1 fM within 1 h of initiation and without cross reaction to 13 porcine virus DNAs. When the RAA-Cas12a-based system was combined with fluorescence, the sensitivity increased to 10 aM. Finally, CORDS can also be freeze-dried in three tubes, making it suitable for transportation and storage, factors that are helpful in resource limited on-site situations. The lyophilized CORDS products are highly stable. Successful detection of ASFV with sensitivity comparable to that of SHERLOCK and without the need for expensive instruments makes our method a robust on-site detection platform.

## Materials and Methods

### Reagents and Instruments

HiScribe T7 Quick High Yield RNA Synthesis kit, Monarch RNA Cleanup Kit, NEBuffer 3.0, EnGen^®^ Lba Cas12a (LbCas12a), and *Ahd*I, *Eco*RI, and *Bam*HI endonucleases were purchased from New England Biolabs (MA, United States). TaKaRa MiniBEST DNA Fragment Purification Kit Ver.4.0 and RNase inhibitor were obtained from TAKARA (Tokyo, Japan). The RAA kit was purchased from Hangzhou ZC Bio-Tech Co., Ltd. (Hangzhou, China). The constant temperature incubator purchased from Senxin (Shanghai, China) was set at 37°C. Fluorescence intensity was measured by the Tecan Infinite M200 plate reader (Tecan, Männedorf, Sweden).

### ASFV Target Design

Thirty-nine ASFV genomes in the National Center for Biotechnology Information (NCBI) database^1^ were analyzed using NCBI BLAST and Perl language programming, from which the ASFV targets were chosen in the conservative regions. The sequences of all the targets obtained were aligned using BLAST with the swine genome (taxid: 9823), porcine reproductive and respiratory syndrome virus (PRRSV), classical swine fever virus (CSFV), and the porcine epidemic diarrhea virus (PEDV) to verify the specificity of the chosen targets. Unspecific targets were removed.

### Nucleic Acid (NA) Preparation

The ASFV p72 gene was synthesized with restriction enzyme cutting sites for *Eco*RI and *Bam*HI. Plasmid pUC57 and the synthesized p72 gene were digested with *Eco*RI and *Bam*HI, and ligation of them was conducted with T4 ligase, thereby producing pUC57-p72 plasmid, which was used as the detection target unless other illustrated.

To prepare the crRNA, the targeted NAs were synthesized along with the T7 promoter and crRNA scaffold, and then cloned into the pUC19 vector by Generay Biotech (Shanghai, China). The transcription templates were prepared by PCR amplification of the plasmid pUC19-target construct. The amplicons were purified using the TaKaRa MiniBEST DNA Fragment Purification Kit (Ver.4.0). The crRNAs were transcribed *in vitro* with the HiScribe T7 High Yield RNA Synthesis Kit. Reactions were performed according to the manufacturer’s instructions for short RNA transcripts in 20 μL volumes at 37°C for 16 h. Transcripts were purified using the Monarch RNA Cleanup Kit and quantified using NanoDrop 1000 (Thermo Fisher, MA, United States). The crRNAs were stored at –80°C.

The custom fluorophore quencher (FQ)-labeled ssDNA reporter (FAM-NNNNNNNNNNNN-BHQ1) for fluorescence assays and FAM-Biotin labeled ssDNA reporter for CORDS assay (FAM-NNNNNNNNNNNN-Biotin) were commercially synthesized by Thermo Fisher.

### Target Cleavage Assays

Briefly, the LbCas12a mediated cleavage targeting assays were performed in 20 μL volumes containing 250 nM LbCas12a, 500 nM crRNA, 0.5 μL RNase inhibitor, 1 × NEBuffer 3.0, and 5 μL linearized pUC57-p72 or non-target dsDNA, which was produced by digestion with *Ahd*I. The reaction was incubated at 37°C for 1 h, and the cleavage products were verified by 1% agarose gel electrophoresis.

### RAA Reactions and Primer Design

Recombinase aided amplification primers were designed for the above-mentioned target using NCBI Primer-BLAST according to the RPA primers design requirements. The primer sequences are listed in Table 1. Primers were synthesized by Thermo Fisher. RAA reactions were conducted using the RAA Kit according to the manufacturer’s instructions in 50 μL volumes containing 41.5 μL buffer A, 400 nM of each RAA primer, 2.5 μL buffer B and 2 μL double-stranded (ds)DNA target at 37°C for 30 min.

**TABLE 1:** DNA and RNA in this study.

**Name**	**Sequence**
ASFV target 1	CATCGGTAAGAATAGGTT
ASFV target 2	AGGATAGAGATACAGCTC
ASFV target 3	TCATAAAATTCTTTTTGC
ASFV target 4	ATGTTTAGGTATTCTGTT
ASFV target 1 crRNA	CAUCGGUAAGAAUAGGUU
ASFV target 2 crRNA	AGGAUAGAGAUACAGCUC
ASFV target 3 crRNA	UCAUAAAAUUCUUUUUGC
ASFV target 4 crRNA	AUGUUUAGGUAUUCUGUU
ASFV target 1 RAA-F	ACATTCATGATTTGCACAAGCCGCACCAAA GCA
ASFV target 1 RAA-R	TGAACATTACGTCTTATGTCCAGATACGTG
ASFV target 2 RAA-F	TATGTAAGAGCTGCAGAACTTTGATGGA
ASFV target 2 RAA-R	AACTAATGTCTGCTCTTAAATGGCC
ASFV target 3 RAA-F	CCACCACACCCGCAGGCTTTCTTCTTGA
ASFV target 3 RAA-R	GTGGCCCGTATTGACGGCAGCATCCCTATG
ASFV target 4 RAA-F	GCTTCTGCCGCTTGAAGCTGTATAAGCATC
ASFV target 4 RAA-R	CTATACGTGAAATTCTTACAATGGA
Forward primer: amplification for T1-crRNA transcription template	GGCTTTACACTTTATGCTTC
Reverse primer: amplification for T1-crRNA transcription template	AACCTATTCTTACCGATGAT

### FAM-BHQ1-Labeled Reporter Assays

FAM-BHQ1-labeled reporter assays contained 250 nM LbCas12a, 500 nM crRNA, 0.5 μL RNase inhibitor, 1 × NEBuffer 3.0, 500 nM FAM-BHQ1-labeled ssDNA reporter and various amount of dsDNA target. The dsDNA targets were pUC57-p72 in a concentration gradient of 1 × 10^–7^, 1 × 10^–9^, 1 × 10^–12^, and 1 × 10^–15^ M. The negative control plasmid lacked sequence homology to the dsDNA targets. Reactions were incubated at a constant temperature of 37°C for 1 h and the fluorescence intensity was measured on a fluorescence plate reader in FAM channel.

For optimizing the concentration ratio of LbCas12a to crRNA in the FAM-BHQ1-labeled assays, reactions were performed as above with the following modifications: the LbCas12a concentration was set at 50 nM; the crRNA concentrations were set at 25, 50, 100, 200, and 300 nM, respectively for ratio gradience; the dsDNA target concentration was 1 × 10^–9^ M.

For the FAM-BHQ1-labeled reporter fluorescence cleavage kinetic assay, the reactions were conducted as described above with the following modifications: the concentration ratio between LbCas12a and crRNA was 50 nM: 100 nM or 250 nM: 500 nM. Reactions were incubated in the fluorescence plate reader for up to 75 min at 37°C with measurements performed every 15 min.

### RAA-Cas12a-Fluorescence Assays

RAA-Cas12a-fluorescence assays include RAA amplification of the dsDNA target and an optimized FAM-BHQ1-labeled reporter assay. The optimized cleavage assay reactions contained the following: 50 nM LbCas12a, 100 nM crRNA, 0.5 μL RNase inhibitor, 1 × NEBuffer 3.0, 500 nM FAM-BHQ1-labeled ssDNA reporter and 2–10 μL RAA reaction. Reactions were incubated at 37°C for 1 h. One-way ANOVA with Dunnett’s post-test set at *p* < 0.05 was used to assess positive results, as was reported previously (Chen et al., 2018).

### CORDS Assays

CORDS assays were conducted using FAM-biotin-labeled ssDNA reporters with commercially available lateral flow strips (Milenia HybriDetect 1, TwistDx; Cambridge, United Kingdom). The assay performs RAA amplification followed by FAM-biotin-labeled ssDNA reporter cleavage with lateral flow strip readout. FAM-biotin-labeled ssDNA reporter cleavage reactions each contained 250 nM LbCas12a, 500 nM crRNA, 0.5 μL RNase inhibitor, 1 × NEBuffer 3.0, 1000 nM FAM-biotin-labeled ssDNA reporter and 5 μL RAA reaction. The reactions were incubated at 37°C for 30–60 min. Then 100 μL of HybriDetect 1 assay buffer was added, and the reactions were run on HybriDetect 1 lateral flow strips. The strips were directly read for band intensity or scanned by Windows Scanner.

The dsDNA targets were diluted in a concentration gradient from 1 × 10^–9^ to 1 × 10^–17^ M to evaluate the assay’s sensitivity. The limit of detection (LOD) was defined as the minimum dsDNA target concentration that generated color development in the test band. Otherwise, the mean m_bc_ and standard deviation σ_bc_ of the gray values on the strips across five replicates were used to determine the positive cut-off values, which were set to the value {m_bc_ + 3σ_bc_}.

To examine the specificity of the developed CORDS assay, 13 nucleic acid samples from porcine viruses were tested. The viruses tested were PRRSV, CSFV, PEDV, epidemic encephalitis B virus (EEBV), transmissible gastroenteritis virus (TGEV), pseudorabies virus (PrV), bovine corona virus (BCoV), porcine rotavirus (PoRV), porcine circovirus (PCV), porcine deltacoronavirus (PDCoV), porcine parvovirus (PPV), foot-and-mouth disease virus (FMDV), and Seneca valley virus (SVV). Whole blood samples with the 13 diseases respectively, were treated with the DNA extraction regent (Cat. # DA-249) purchased from DAAN Gene Co., Ltd., of Sun Yat-sen University (Guangzhou, China). Briefly, 450 μL DNA extraction regent was added to 200 μL blood sample. Then the mixture was mixed well and spun shortly for 5–10 s. The mixture was incubated at 100°C for 10 min, and then centrifuged at ≥10000 rpm for 2 min. The supernatant was the nucleic acid sample for CORDS assay.

### Lyophilized CORDS Assays

The lyophilized CORDS assay has three tubes: the RAA mix (tube A), RAA buffer mix (tube B), and Cas mix (tube C). The RAA buffer mix contains 400 nM of each primer, as well as RAA buffer A and RAA buffer B for a 50 μL reaction. The Cas mix contains FAM-biotin-labeled ssDNA reporter cleavage reactions of CORDS assay mentioned above. The RAA buffer mix and Cas mix were pre-frozen at −80°C for 1–3 h and then freeze-dried at −50°C overnight, respectively. The lyophilized RAA mix (tube A) was supplied with the RAA kit.

Lyophilized CORDS assay, which was conducted by first dissolving the lyophilized powder, involved dissolving tube A into a 50 μL reaction containing dsDNA target. The solution was then transferred to tube B, incubated at 37°C for 30 min. Then 2–10 μL of the RAA reaction was added to tube C and diluted into a reaction volume of 20 μL. The reactions were incubated at 37°C for 30–60 min. Then 100 μL of HybriDetect 1 assay buffer was added, and the reactions were run on HybriDetect 1 lateral flow strips. The strips were directly read for band intensity or scanned by Windows Scanner.

To evaluate the stability of the lyophilized CORDS assay, the individual tubes (tube B) were incubated at 37°C for 24, 72 or 168 h before verification. Each tube C was stored at 4°C for 168 h before verification.

## Results

### Cas12a Cleavage Activity Guided by crRNA

**FIGURE 1:**
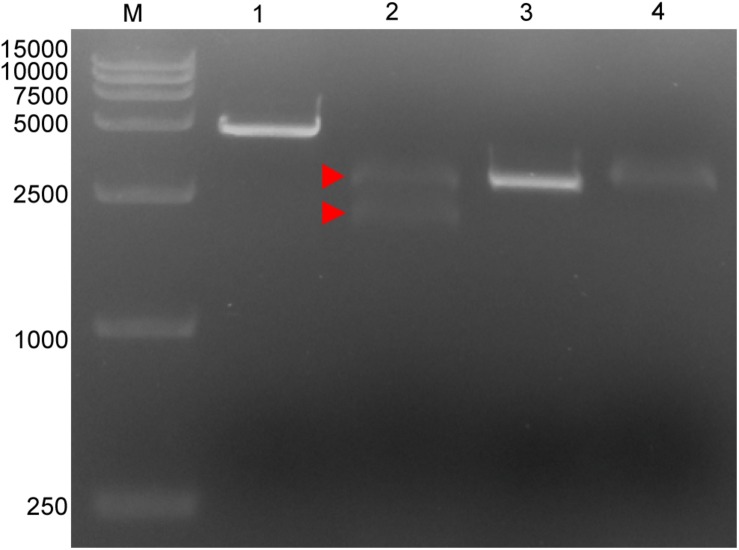
Cleavage activity of crRNA-guided LbCas12a. M: DL15000 DNA marker; 1: intact linearized target dsDNA (4618 bp); 2: cleavage products from linearized target dsDNA; 3: intact linearized non-target dsDNA (2966 bp); 4: linearized non-target dsDNA after cleavage reaction. dsDNA binding to the LbCas12a-crRNA complex is marked by a red triangle where the band can be seen to shift upward compared with the intact linearized dsDNA. The expected cleavage products are marked by red arrows.

Four targeted sequences were found by NCBI BLAST and Perl language programming in the conservative regions. The target sequences are listed in Table 1. Based on these sequences, crRNAs were designed and prepared via *in vitro* transcription (Table 1). Target 1, which is located in the p72 gene, was used for subsequent tests. The *in vitro* cleavage results confirmed the crRNA guided LbCas12a cleavage activity of the assay (Figure 1).

### The Cas12a-Based ASFV Nucleic Acid Fluorescence Reporting System

**FIGURE 2:**
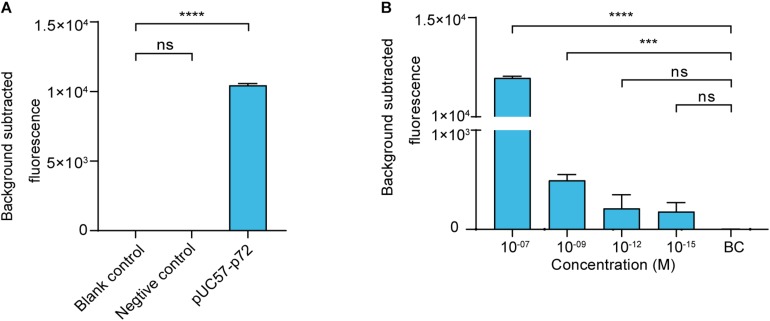
Cas12a-based ASFV NA fluorescence reporting system. **(A)** Validation of the Cas12a-based ASFV nucleic acid fluorescence reporting system. The concentrations of the negative control (i.e., non-target dsDNA used in Figure 1) and pUC57–p72 were both 1 × 10^– 8^ M. **(B)** Sensitivity of the Cas12a-based ASFV NA fluorescence reporting system. Background subtracted fluorescence was the fluorescence intensity of the experimental group against the blank control. BC means blank control group in which targeted nucleic acid was substituted by DNase-free water. Error bars in panels **(A,B)** represent the mean ± SD, where *n* = 3 replicates. ^∗∗∗^*p* ≤ 0.001 and ^∗∗∗∗^*p* ≤ 0.0001.

We then established a fluorescence reporting system based on the cleavage activity of LbCas12a. To evaluate its validity, a negative control and pUC57–p72 were separately added to the system as targets. The statistical analysis revealed that there was no significant difference (*p* > 0.05) between the blank control and the negative control groups. The pUC57–p72 group showed significantly higher fluorescence intensity than the other groups (*p* < 0.0001). These results confirm that crRNA-complementary dsDNA was able to trigger LbCas12a to cleave the FAM-BHQ1-labeled ssDNA reporter (Figure 2A). The reporting system had sufficient sensitivity to detect pUC57–p72 at a concentration of 1 × 10^–9^ M (Figure 2B).

### Establishing the RAA-Cas12a-Fluorescence Assay

**FIGURE 3:**
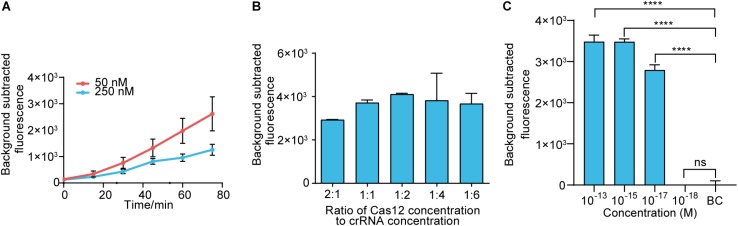
Establishing the RAA-Cas12a-fluorescence assay. **(A)** Fluorescence kinetics at different LbCas12a concentrations. **(B)** Optimizing the concentration ratio of LbCas12a to crRNA. **(C)** Sensitivity of the RAA-Cas12a-fluorescence assay. Background subtracted fluorescence was the fluorescence intensity of the experimental group against the blank control. BC means blank control group in which the template of RAA amplification was substituted by DNase-free water. Error bars in panels **(A–C)** represents the mean ± SD, where *n* = 3 replicates. ^∗∗∗∗^*p* ≤ 0.0001.

Before assay establishment, we optimized the concentration of LbCas12a by measuring the fluorescence intensity kinetics of LbCas12a targeting. The 50 nM group showed a more obvious difference in fluorescence intensity than the 250 nM group (Figure 3A). Thus, the concentration of LbCas12a was set at 50 nM for the fluorescent assays in subsequent experiments. We also investigated the appropriate concentration ratio of LbCas12a to crRNA. The group with a 1:2 ratio showed better performance and reproducibility than the others (Figure 3B). The optimized system was combined with RAA to establish the RAA-Cas12a-fluorescence assay. The LOD for the assay reached 10 aM (Figure 3C).

### Establishing the CORDS Assay

**FIGURE 4:**
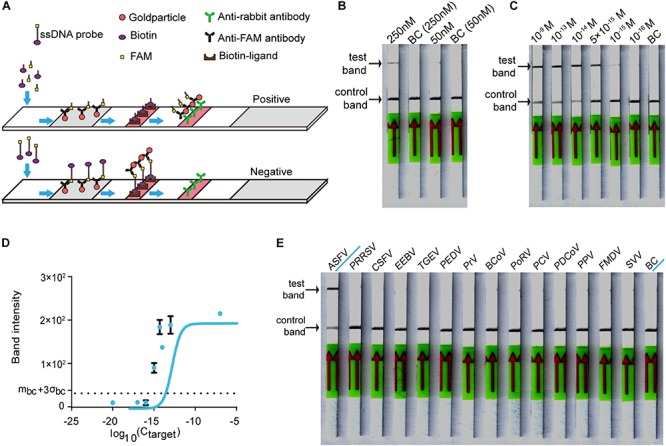
Establishing the CORDS assay. **(A)** Schematic diagram of the CORDS assay. **(B)** Optimization of the concentration of LbCas12a. **(C)** Sensitivity of the CORDS assay. **(D)** Mean gray values of the test band at different dsDNA target concentrations. The dashed line m_bc_ + 3σ_bc_ indicates the positive cut-off. **(E)** Specificity of the CORDS assay. BC: blank control.

For on-site detection, we substituted the fluorescence intensity readout in the RAA-Cas12a-fluorescence assay with lateral flow strips to establish the CORDS assay (Figure 4A). To determine the appropriate concentration of LbCas12a for the CORDS assay, we next compared two groups in which the concentrations of LbCas12a were 250 and 50 nM. The former showed a more obvious test-band intensity difference between the experiment group and the blank control (Figure 4B). Therefore, we chose 250 nM as the concentration of LbCas12a for the CORDS assays, and the dsDNA targets were diluted from 1 × 10^–9^ M to 1 × 10^–17^ M for sensitivity evaluation. The LOD of the assay was 1 × 10^–15^ M according to the concentration gradient test results (Figure 4C). The gray values of the test bands in the CORDS assays were measured and recorded. A positive result was recorded when the concentration of the dsDNA target was 1 × 10^–15^ M, which further confirmed the assay’s sensitivity (Figure 4D). The 13 NA samples from typical porcine viruses were tested with this assay, all of which were negative. Thus, no cross-reactions were observed (Figure 4E).

### Assessing the Stability of the Lyophilized CORDS Assay

**FIGURE 5:**
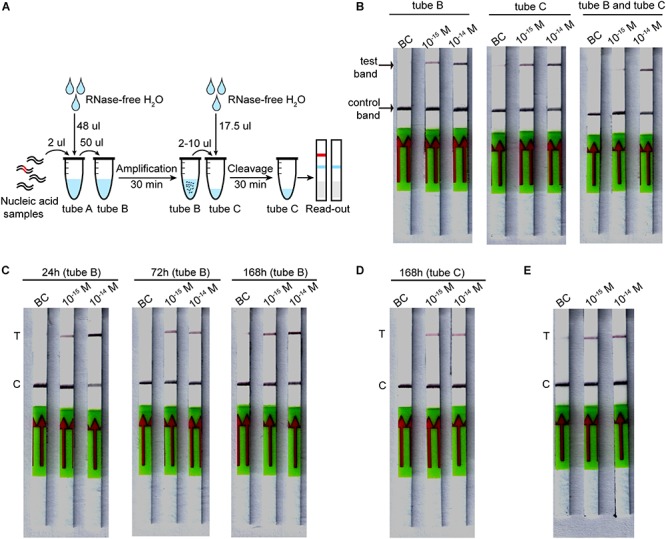
Lyophilized CORDS assay. **(A)**. Workflow for the lyophilized CORDS assay. **(B)** Verification of tubes B and C. **(C)** Accelerated stability test on tube B at 37°C. **(D)** Accelerated stability test on tube C at 4°C. **(E)** Reduction of the incubation time for LbCas12a cleavage. T and C in panels **(C–E)** represents the test and control bands, respectively.

The components of the CORDS assay were lyophilized in three tubes to simplify the workflow (Figure 5A). The lyophilized RAA buffer mix (tube B) and Cas mix (tube C) were verified and the two tubes were tested for further confirmation. The results illustrated that the LOD was still 1 × 10^–15^ M (Figure 5B). Accelerated stability tests were performed on tubes B and C. Tube B was still functional at a target concentration of 1 × 10^–15^ M after storage at 37°C for 168 h (Figure 5C). Tube C still worked well after storage at 4°C for 168 h without any sensitivity loss (Figure 5D).

## Discussion

Since August 2018, ASF has affected 32 provinces in China and caused great economic losses (Food and Agricultural Organization of the United Nations, 2019). ASF occurred as a rapid outbreak across the nation, indicating its highly contagious nature (Food and Agricultural Organization of the United Nations, 2019). Given its similar clinical signs to several other porcine diseases (Galindo and Alonso, 2017) and the lack of laboratory facilities for diagnosis on farms, diagnosing ASF often requires infected samples to be transported to a qualified laboratory, which delays diagnosis and increases the chances of contagion. Hence, accurate and on-site diagnosis of ASF is essential for surveillance and control of ASF outbreaks.

Ours is the first report of a Cas12a-based assay using immunochromatographic lateral flow strips, termed CORDS, which enables the on-site detection of ASFV in 1 h (Figure 5E). This new assay requires no instrumentation other than a 37°C incubator, and its reaction temperature (body temperature) could be easily attained in an on-site situation. The whole reaction system comprises three tubes of lyophilized powder. Thus, the CORDS assay’s workflow only includes dissolution of lyophilized powders and addition of the samples, without any complex technical requirements. The whole detection process takes only 1 h and displays its result through the band visualized on a lateral flow strip. Therefore, the CORDS assay has the advantage of detecting ASFV on-site (i.e., on farms or in slaughter houses) in a short time and without access to a diagnostics laboratory.

As a highly specific lateral flow strip-based assay, the specificity of CORDS is guaranteed by LbCas12a-crRNA targeting. LbCas12a-crRNA can discriminate single nucleotide mutations such as point mutations in the seed region (Chen et al., 2018; Santos et al., 2018). In the present study, the CORDS assay did not cross-react with 13 nucleic acid samples from typical porcine viruses, making it superior to the methods reported until now (Gao et al., 2018; Miao et al., 2019). Thus, CORDS can distinguish ASF from other porcine diseases sharing similar clinical signs easily on site, even avoiding confusion between CSF and PRRS.

The LOD of the CORDS assay is at the femtomolar level for ASFV-specific NAs. To be precise, the system can detect ASFV p72 gene copies as low as 6 × 10^5^ copies/mL, which is more sensitive than isothermal amplification-based assays (Oura et al., 2013) (which are considered good choices for on-site viral detection) and compares well with the newly reported lateral flow strip based assays for ASFV detection (Gao et al., 2018), Miao et al., 2019). The sensitivity of this assay is sufficient for ASF diagnosis on farms or in slaughter houses. During the ASFV infection process, blood virus levels can increase to 1 × 10^6^ copies/mL in 5–8 days post-infection (dpi) (Olesen et al., 2018). ASFV infections can result in virus levels of up to 1 × 10^9^ TCID_50_/mL in the blood (Dixon et al., 2019), indicating that virus genome copies can achieve >1 × 10^9^ copies/mL. Hence, the femtomolar sensitivity of the CORDS assay should diagnose ASF at an early stage of infection (5–8 dpi) when the virus genome copy number reaches 1 × 10^6^.

For ease of use, the CORDS assay components are lyophilized in three tubes: the RAA mix, RAA buffer mix, and the Cas mix. We found that the assay maintained femtomolar sensitivity after lyophilization. Lyophilization significantly simplifies the operational process and improves the detection stability. According to our accelerated stability tests, the RAA buffer mix still maintained femtomolar sensitivity after storage at 37°C for 1 week. The lyophilized Cas mix should work well after storage at 4°C for 1 week without any sensitivity decrease. Therefore, these stability tests show that CORDS can be stored and transported under the conditions provided by the diagnostic kit’s manufacturers. It is envisaged that lyophilized CORDS has potential for industrial manufacturing and widespread on-site application. According to the accelerated stability tests, the lyophilized Cas mix is not as stable as the lyophilized RAA buffer mix and manufacturer provided RAA mix. Considering Cas12a is a rather stable protein, the cause might be instability of the crRNA. RNA modification or capping may help to solve this problem and enable transportation at room temperature to lower the assay costs.

In addition to the lateral flow-strip readout, the RAA-Cas12a-based system also provides a fluorescent readout assay with high sensitivity. Our Cas12a-based nucleic acid fluorescence reporting system reached a sensitivity level of 1 × 10^–9^ M without amplification of dsDNA targets. In combination with RAA amplification, the RAA-Cas12a-fluorescence assay detected the dsDNA target at a sensitivity level of 10 aM, thereby reaching the level of other Cas12/13-based assays (Gootenberg et al., 2017, 2018; Chen et al., 2018; Li et al., 2018; Myhrvold et al., 2018; Santos et al., 2018). The fluorescent readout format also enables kinetic tests on reporter cleavage, providing information for reaction optimization and the possibility of more accurate detection. Compared with the well-established real-time PCR, the RAA-Cas12a-fluorescence system is capable of high-throughput detection with lower costs and simpler operation.

To sum up, RAA-Cas12a-based system, especially the CORDS assay, is a promising ASFV detection method with high sensitivity and specificity, as well as having no need for scientific instruments and skilled operators. The lyophilized CORDS assay is now ready for industry manufacturing and wide-ranging applications.

## Data Availability Statement

The raw data supporting the conclusions of this article will be made available by the authors, without undue reservation, to any qualified researcher.

## Author Contributions

LH, JB, HSL, and HJL contributed conception and design of the study. JB and HSL performed the experiments and wrote the manuscript. YZ and JL performed the fluorescence experiments. GZ and YZ analyzed the data. LW and WJ made figures. HD, JY, and QX advised on experimental design and data interpretation. LH supervised the study, interpreted the data, wrote the manuscript and acquired the research funds.

## Conflict of Interest

The authors declare that the research was conducted in the absence of any commercial or financial relationships that could be construed as a potential conflict of interest.

## Footnotes

1 https://www.ncbi.nlm.nih.gov/
